# Predicting Coral Recruitment in Palau’s Complex Reef Archipelago

**DOI:** 10.1371/journal.pone.0050998

**Published:** 2012-11-28

**Authors:** Yimnang Golbuu, Eric Wolanski, Jacques Wasai Idechong, Steven Victor, Adelle Lukes Isechal, Noelle Wenty Oldiais, David Idip, Robert H. Richmond, Robert van Woesik

**Affiliations:** 1 Palau International Coral Reef Center, Koror, Palau; 2 School of Marine and Tropical Biology, James Cook University, Townsville, Australia; 3 Australian Institute of Marine Science, Townsville, Australia; 4 Nature Conservancy, Palau Field Office, Koror, Palau; 5 Kewalo Marine Laboratory, University of Hawaii at Manoa, Honolulu, Hawaii, United States of America; 6 Department of Biological Sciences, Florida Institute of Technology, Melbourne, Florida, United States of America; University of Texas, United States of America

## Abstract

Reproduction and recruitment are key processes that replenish marine populations. Here we use the Palau archipelago, in the western Pacific Ocean, as a case study to examine scales of connectivity and to determine whether an oceanographic model, incorporating the complex reef architecture, is a useful predictor of coral recruitment. We tested the hypothesis that the reefs with the highest retention also had the highest densities of juvenile coral density from 80 field sites. Field comparisons showed a significant correlation between the densities of juvenile *Acropora* colonies and total larval recruitment derived from the model (i.e., calculated as the sum of the densities of larvae that self-seeded and recruited from the other reefs in the archipelago). Long-distance larval imports may be too infrequent to sustain coral populations, but are critical for recovery in times of extreme local stress.

## Introduction

**Figure 1:**
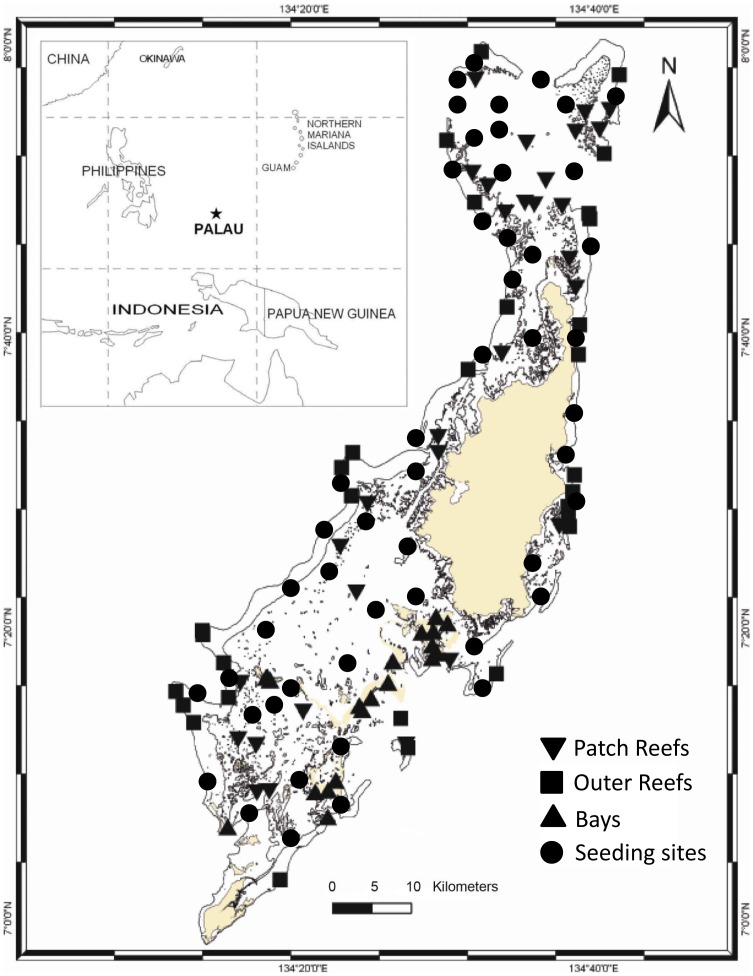
Eighty study sites surveyed in Palau, 2009. The study region was stratified into three habitats, as either inner reef bays (n = 20), patch reefs (n = 30), or outer reefs (n = 30). Dark circles indicate seeding sites. The land is depicted in yellow.

Marine connectivity is defined as the sharing of a gene pool through the process of larval dispersal and settlement. The broadcasting of gametes and planktonic larvae are the most common means of marine dispersal. Identifying the extent of larval exchange among marine ecosystems is of primary importance for furthering our understanding of connectivity among marine populations [1], [2]. Early coral-reef studies suggested that larval exchange among coral reefs occurred at large, regional scales [3], [4]. More recent oceanographic models for the Caribbean and elsewhere have suggested that larval connectivity is unlikely, or rare, at scales of hundreds of kilometers or more [2]. In support of the model outputs, recent genetic studies suggest that most larval exchange is local, at the scale of 1–10 km [5]–[8]. Here we use Palau (Figure 1) as a case study to examine local scales of larval connectivity, to determine whether coral recruitment was predictable, both through self-seeding and connectivity, and to discuss the implications of the results in the context of maintaining reef resilience.

**Figure 2:**
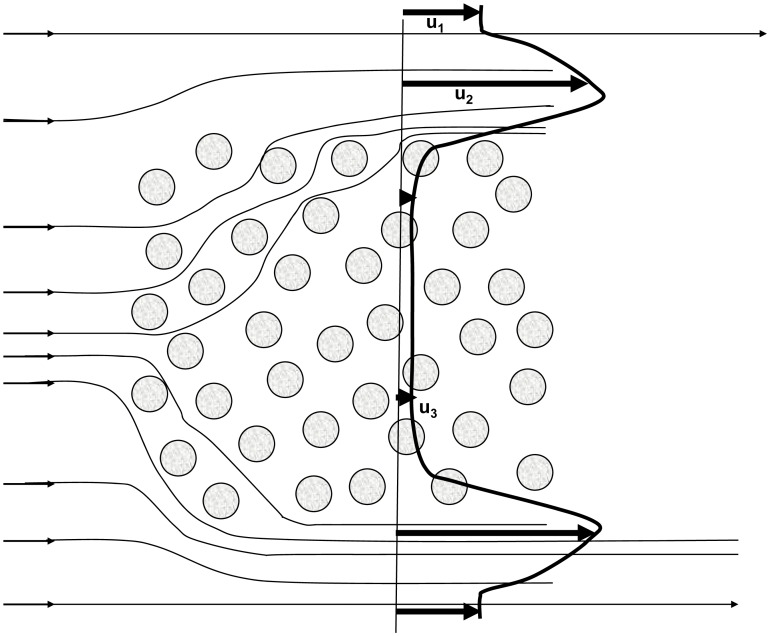
A schematic of the ‘sticky-water’ effect in a reef system under a steady current. The circles represent reefs, the thin lines represent streamlines of the mean currents, and the thick line is the distribution of the mean currents along a transverse section, extending from the surrounding waters in through the reefs and out to the surrounding waters. The penetration of water circulation inside the archipelago is asymmetric, being larger on the upstream side than on the downstream side. The thin, straight vertical line is a transverse section, inserted for comparative purposes, to show the different vectors for u_1_, u_2_, and u_3_. The sticky water effect generates slower mean currents (u_3_) inside the archipelago compared with currents surrounding the archipelago (u_1_). The deflection of the mean circulation around the archipelago also generates a boundary layer where the currents are even faster than elsewhere (u_2_), so that u_2_> u_1_> u_3_.

Oceanographic studies of self-seeding and hydrodynamic connectivity among reefs have focused on oceanic or tidal eddies, generated by flow around bathymetric features, which trap water-borne larvae [2], [9]–[21]. These studies have focused on isolated islands. Water circulation in and around the Palau archipelago is very different, however, from circulation around an isolated island or reef, because the mean-water circulation is steered away from and around the archipelago by a phenomenon called the ‘sticky-water’ effect (Figure 2) [22]. This sticky-water effect generates slower mean currents (u_3_) inside the archipelago compared with currents surrounding the archipelago (u_1_). The deflection of the mean circulation around the archipelago also generates a boundary layer where the currents are even faster than elsewhere (u_2_), so that u_2_> u_1_> u_3_. The sticky-water effect is also enhanced in shallow waters (depth <20 m) by the non-linear friction-driven interaction between the tidal currents and the mean currents [24]. In theory, the slow mean currents inside the archipelago should enhance self-seeding within the reef complex [23], but no studies have been carried out on the connectivity among reefs in the reef mosaic of Palau.

**Figure 3:**
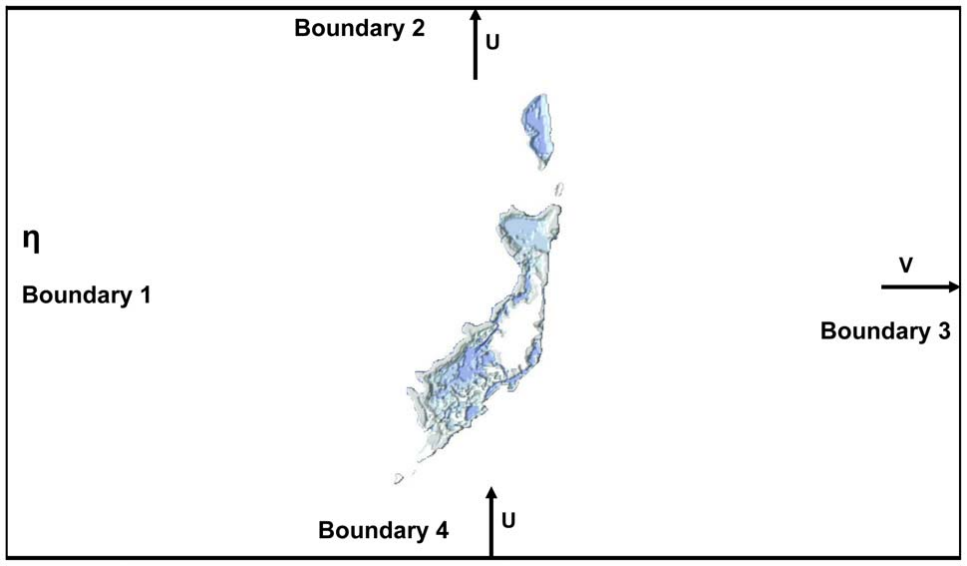
The model domain and the open boundary conditions in the Palau archipelago; the tides η were specified on the western boundary (boundary 1). The north/south current U (>0 if northward, <0 if southward) was specified both on the northern boundary (boundary 2) and southern boundary (boundary 4).The east/west current V (>0 if eastward, <0 if westward) was specified on the eastern boundary (boundary 3).

To test whether coral recruitment was predictable in Palau we developed a coral larval oceanography model for the archipelago and the surrounding ocean (Figure 3). This model provided the hydrodynamics data needed to track waterborne larvae using advection-diffusion equations. The model simulated the fate of coral larvae after a spawning event, and estimated the probabilities of both self-seeding and connectivity among localities. These data were then compared with coral cover and juvenile coral densities collected in the field. We tested the hypothesis that the reefs with the highest retention also had the highest densities of juvenile corals. This information will be useful to establish conservation priorities. There is a national effort in Palau to establish a Protected Areas Network, which may provide regional resilience to both local and global scale stressors. But currently there are limited data to guide selection of sites for the network. Our study is an important step toward filling this information gap.

## Results

**Figure 4:**
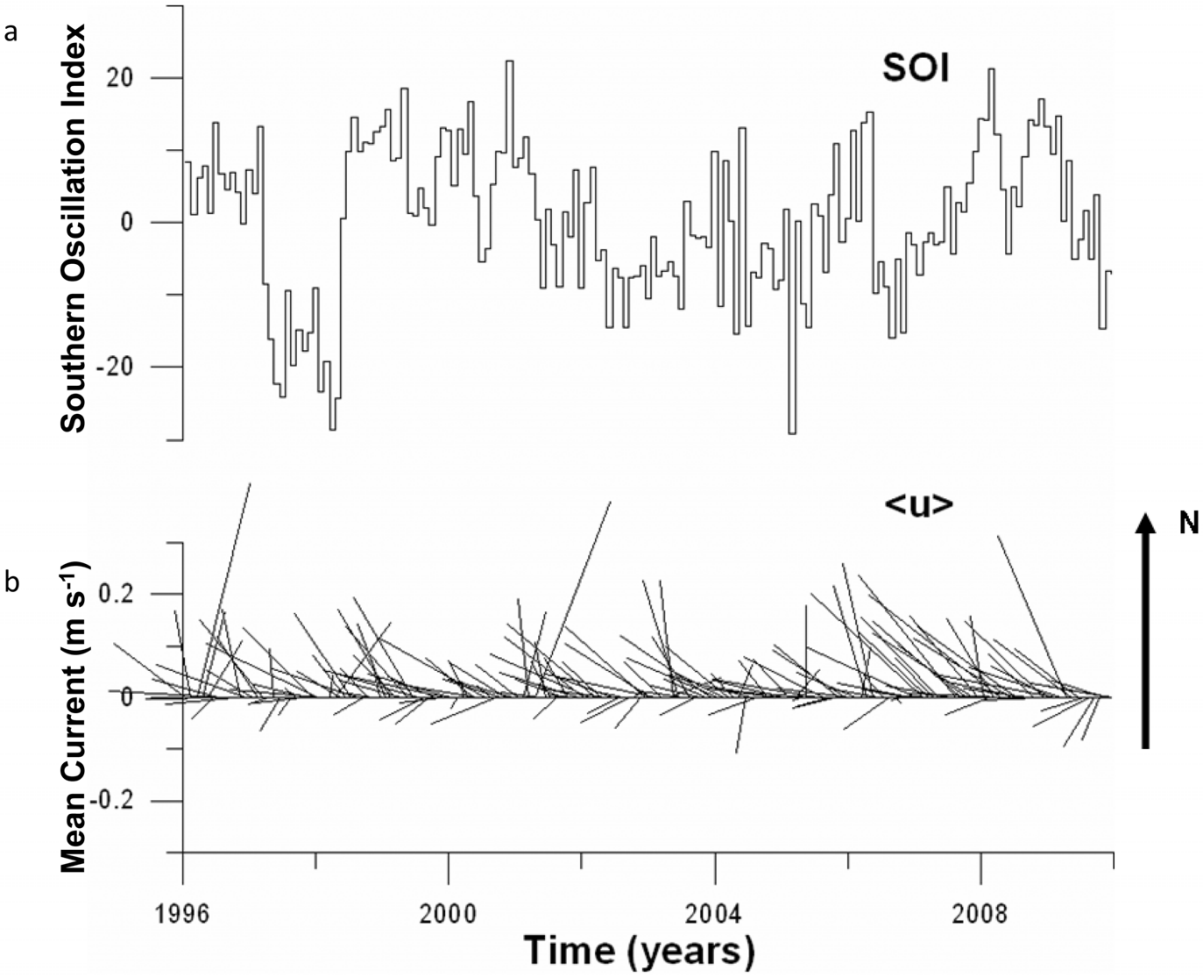
(a) Time-series stick plot of the Southern Oscillation Index (SOI) and (b) the altimetry-derived, near surface, monthly averaged currents <u>, indicating direction and magnitude (length of vectors) in the waters off the Palau archipelago.

The altimetry-derived, long-term average, surface currents in the ocean near Palau were from southeast to northwest, at a speed of about 0.12 m s^−1^. These currents also fluctuated through time, being generally the fastest (∼0.25 m s^−1^) during La Niña years and the slowest (∼ 0.05 m s^−1^) during El Niño years (Figure 4). Fast and slow monthly mean currents occurred as events that were independent of the Southern Oscillation Index (SOI). Indeed, there was no significant correlation between the SOI and the monthly averaged currents off Palau (Figure 4). This independence is a consequence of Palau’s location in the shear zone between the eastward-flowing North Equatorial Counter Current, to the south of Palau, and the westward-flowing North Equatorial Current, to the north of Palau. The resultant surface currents around Palau are dominated by transient, oceanic eddies [25].

The altimetry data also showed that water-borne larvae from coral reefs around Yap could drift to Palau in about 45 days. Using 168 months of data, there were three occasions in the last decade or more when a coral larvae ‘connection’ between Yap and Palau was highly probable: July and August 1997, August 1999, and August and September 2006. But coral spawning occurs twice a year in Micronesia, in April – May and again in August-September, over a period of 4 months [26]. In this regard, the oceanography suggests that during the spawning seasons, the corals on reefs around Yap could potentially seed Palau reefs 9% of the time (i.e., 4 months out of the 42 months [168/4] or about 1 year in every 11 years).

**Figure 5:**
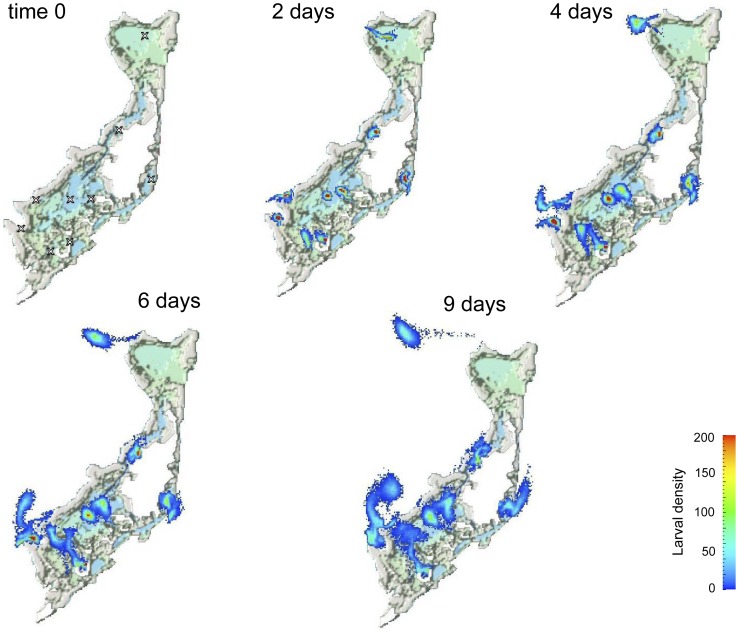
An example of virtual coral larvae dispersal plumes 0, 2, 4, 6, and 9 days after coral spawning in the Palau archipelago. The maximum concentration was arbitrarily set at 200 for visualization purposes.

Reef density in the northern lagoon was low, at 26%, compared with 41% in the southern lagoon. Such differences in reef density formed the sticky-water effect in the southern lagoon. The stick-water effect was not apparent in the northern lagoon (Figure 5). The resulting mean residence time (i.e., the time for the particle concentration to decrease by 64%, i.e., a factor of 1/*exp*) was 14 days in the northern lagoon and 47 days in the southern lagoon.

**Figure 6:**
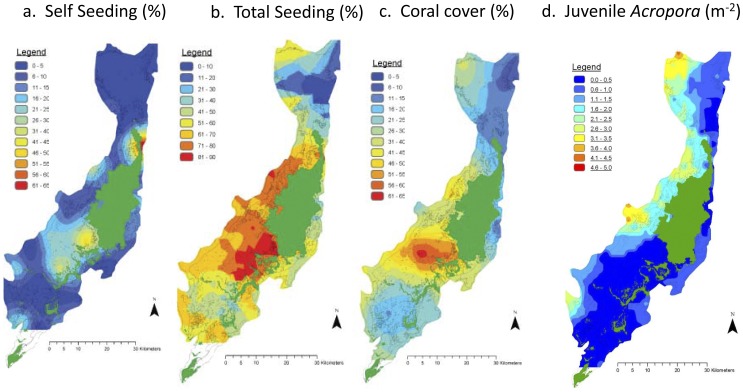
Distribution of (a) self-seeding rate (%), (b) total seeding rate (%), (c) coral cover (%) and (d) juvenile *Acropora* density (m^2^)in the Palau archipelago.

The model showed that the average self-seeding potential across Palau’s main archipelago was about 10%. Five days after simulated spawning, retention values ranged from 0 to 66% across all sites. The highest retention (Figures 6a and 6b) was localized within the northern portion of the southern lagoon, where reef density was the highest. The western side of the archipelago also exhibited retention comparable to the northern section of the southern lagoon. The fringing and barrier reefs on the eastern side of the main island of Babeldaob showed relatively high retention, with values comparable with the sites on the western side of Babeldaob. The southern lagoon was also a high retention area, except in the reef passages and over the reef flat, where the flow rates were consistently high and retention was low. The lowest retention values were evident in the northern lagoon, where reef density was low and flow was predominantly east to west (Figure 6).

**Figure 7:**
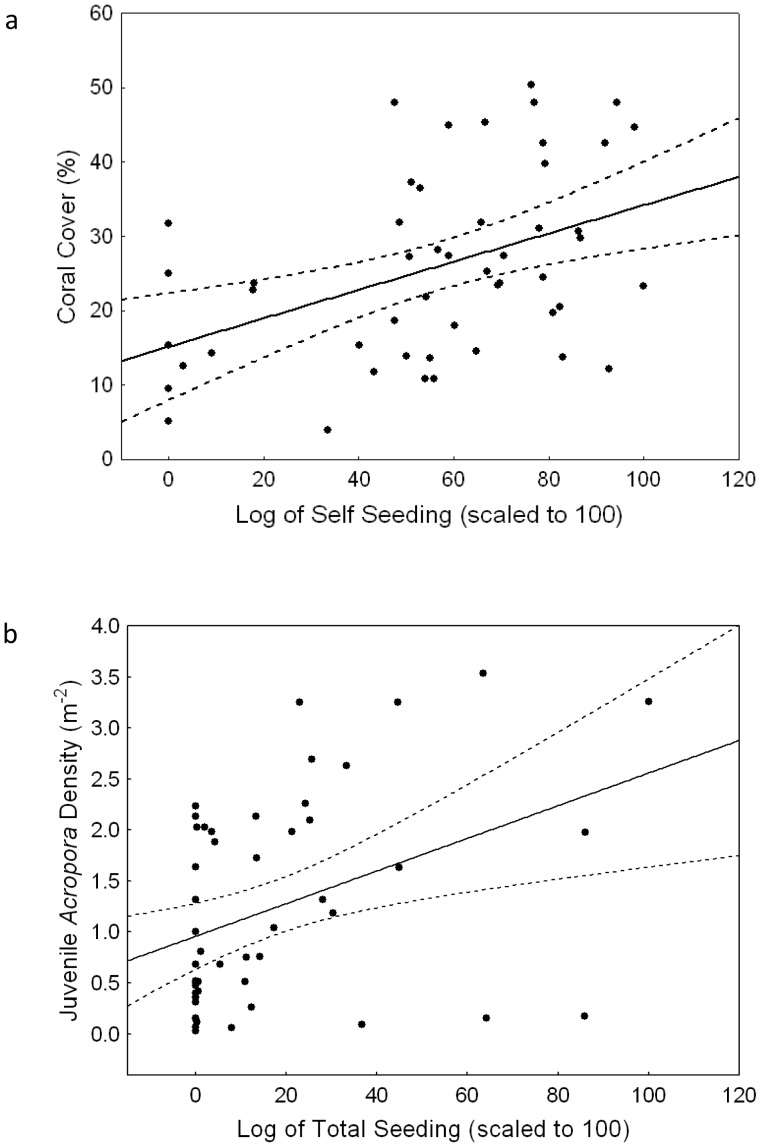
(a) The relationship between self-seeding, as predicted by the oceanographic model, and coral cover that was measured in the field. The dark line indicates the linear response following the function, *y* = 15.8+0.19*x* (*R* ^2^ = 0.19), the dashed lines indicate the 95% confidence intervals, and the dark circles represent the data. (**b**) The correlation between total seeding, as predicted by the oceanographic model, and juvenile *Acropora* density that was measured in the field. The dark line indicates the linear relationship, the dashed lines indicate the 95% confidence intervals, and the dark circles represent the data.

Within the Palau archipelago, there was a significant relationship between the density of self-seeding, estimated by the oceanographic model, and the percentage of coral cover estimated in the field (Figures 6c and 7, *_R_* ^2^ = 0.19, p = 0.002). There was no significant correlation between sites that self-seeded and juvenile *Acropora* colonies (p = 0.30). The lack of correlation between self-seeding, derived from the model, and the densities of juvenile *Acropora* is most likely because self-seeding and recruitment from elsewhere were indistinguishable in the field. There was, however, a significant correlation between the density of juvenile *Acropora* colonies and the total density of larval recruits that were derived from the model, calculated as the sum of self-seeding and larvae from other sites (Figure 6d, r_s_ = 0.29, p = 0.03). There were no significant correlations with sites that primarily received larvae from other sites and coral cover. There were also no significant correlations between juvenile brooding corals and predicted retention sites. The Moran’s I test for spatial autocorrelation showed no spatial autocorrelation of juvenile *Acropora* colonies on outer reefs (Moran’s I = 0.01), but, as expected, showed some spatial autocorrelation in the lagoon (Moran’s I = 0.09).

## Discussion

The hydrodynamic model showed considerable local retention at all sites in the Palau archipelago for time scales of three days and less. But at longer time scales, the northern reefs were well flushed and the simulated larvae were lost, whereas the southern lagoon retained larvae (Figures 5 and 6). Several studies have shown that larval behavior influences recruitment [27], [28], but only at the scale of centimeters to meters. Therefore, we did not incorporate larval behavior in our model because we were interested in the hydrodynamics and the larval retention capacity at the scale of kilometers. The results indicated that both self-seeding (auto-seeding) of reefs and recruitment from other reefs (allo-seeding), by waterborne dispersal of larvae, is common within the Palau archipelago. The former prevails in areas of high-reef density (i.e., in the southern lagoon), and the latter is most common in areas of low-reef density (i.e., in the northern lagoon).

The reefs in the eastern central lagoon near Babeldaob, which displayed high (simulated) larval retention, were not in good condition and supported fewer juvenile *Acropora* colonies than other high-retention areas. The poor condition of reefs in the eastern central lagoon may be related to a combination of substrate instability [29] and considerable terrestrial discharge onto these reefs from adjacent watersheds [30]–[32]. Therefore, our study also supports the fact that coral populations are only self-sustaining if terrestrial discharge is controlled [29], [32].

The highest retention was apparent in the southern lagoon where reef density was also high. Such high retention was not apparent in the northern lagoon, where reef density was low. We found a positive correlation between the observed density of juvenile *Acropora* colonies and predicted larval recruitment calculated as the sum of self-seeding and imports from other reefs. Therefore the model was a reasonable predictor of juvenile *Acropora* colonies. There was also a significant relationship between the density of self-seeding predicted by the model and the percentage of coral cover measured in the field. Coral cover is, however, influenced by multiple variables and not just, simply, larvae supply and local hydrodynamics. Studies using genetic techniques to assess the relative importance of differential fragmentation are needed to provide further insight on other processes that may also contribute to differential coral cover.

**Figure 8:**
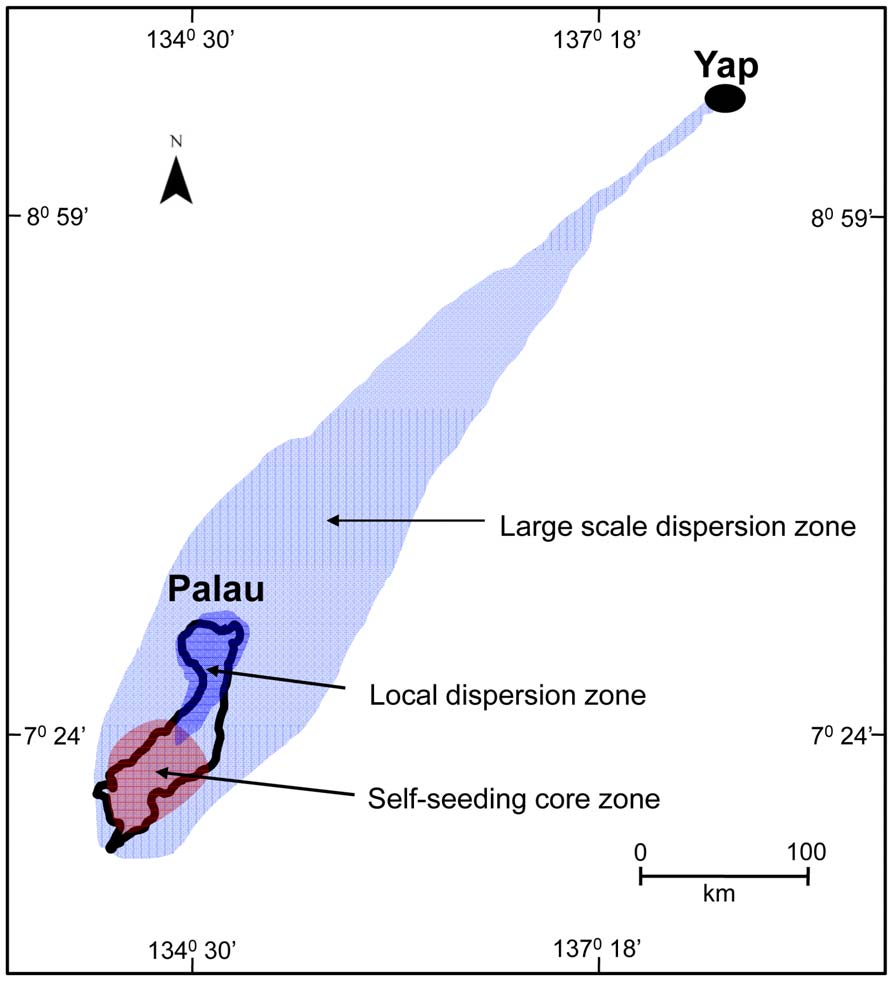
The three zones of coral larvae connectivity identified within the Palau archipelago include: (i) the large scale dispersion zone (depicted in light blue), providing infrequent (decadal) connectivity between Yap and the Palau archipelago, (ii) the local dispersion zone (depicted in purple), and (iii) the self-seeding core zone (depicted in red).

On ecological time scales, coral populations in Yap could feasibly connect with coral populations in the Palau archipelago, given that scleractinian coral larvae can remain competent for over 100 days [33], [34]. Although the mean oceanic currents in the region are primarily towards the northwest, these currents are variable and occasionally flow southwestward (Figure 8). The altimetry data showed that water-borne larvae from coral reefs around Yap could drift to Palau in about 45 days and coral reefs around Yap could potentially seed Palau reefs 9% of the time (i.e., about 1 year in every 11 years). Therefore, Palau reefs are potentially self-sustaining, but may receive occasional recruitment pulses from Yap on a temporal scale of once a decade. Long-distance dispersal from Yap most likely occurred in August 1999, one year after the thermal-stress event in 1998 caused widespread coral mortality in Palau. Notably the corals in Yap did not undergo major thermal stress in 1998. This long-distance dispersal event may have facilitated the re-establishment of corals in Palau.

These general results agree with contemporary genetic studies that suggest that most recruitment is local but there is occasional, long-distance connectivity ensuring panmictic populations [5], [6], [8]. Therefore, connectivity at the scale of hundreds of kilometers (i.e., from Yap to the Palau archipelago) does not occur often; however connectivity is likely at least once a decade, in view of the major circulation patterns (Figure 3). This occasional connectivity may be ecologically critical to enable coral populations to recover following major mortality events, such as the large-scale coral bleaching event that occurred during the 1998 El Niño [8], [35].

In general, this study shows that recruitment was apparent at three spatial and temporal scales: (i) local and yearly self-seeding, which was potentially enhanced by a high-reef density, (ii) archipelago-wide, yearly recruitment through waterborne dispersal of seeds originating from a high-density reef matrix, and (iii) regional, decadal recruitment of larvae after long-distance waterborne dispersal from reefs potentially hundreds of kilometers away (Figures 5, 6 & 8).

This study suggests that the reefs of Palau are highly connected, which supports the concept of establishing and strictly enforcing networks of Marine Protected Areas that include No-Take Areas. Coral populations may be maintained by a network of Marine Protected Areas in the high density core zone, in the dispersion zone downstream of the core zone, and also, ideally, in the long distance source zone. Such a strategically located network is urgently needed in Micronesia to maintain connectivity and sustain the marine populations throughout the archipelago. Larval ‘imports’, from other nations may be too infrequent to sustain local coral-reef systems, but those larvae are critical for recovery in times of extreme stress [35]. Designing resilient networks of conservation areas may be also the best defense against future climate-change disturbances.

## Materials and Methods

### Ethics Statement

All research was approved by the Palau International Coral Reef Center. The Ministry of Natural Resources, Environment, and Tourism of the Republic of Palau provided the permit to conduct this research.

### Study Location

The Palau archipelago (07° 30' N, 134° 30' E) is located in western Micronesia (Figure 1). The archipelago consists of the volcanic island of Babeldaob (332 km^2^) and smaller atolls, coral platform islands, and raised limestone islands. The archipelago is about 700 km long and the islands are surrounded by barrier, fringing, and patch reefs that make up a lagoon, which together cover a surface area of approximately 1,450 km^2^. The archipelago is located in a high shear zone between the westward-flowing North Equatorial Current to the north and the eastward-flowing North Equatorial Counter Current to the south. The field survey sites and the virtual seeding sites were focused in the main archipelago around Babeldaob Island and its fringing reefs to the north and south, including the high-density reef mosaic to the south that includes numerous reefs and raised limestone islands known as the Rock Islands.

### Field Surveys

A total of 80 sites were surveyed for coral cover and juvenile coral density in 2009 (Figure 1). The sampling area was stratified by habitat as: (i) bays, (ii) patch reefs, and (iii) outer reefs, using the 2005, National Oceanic and Atmospheric Administration (NOAA) derived shallow benthic habitat maps of Palau [36]. The habitat shapefiles were accessed using Arc9.3® from which random points were selected, using the Hawth’s Analysis Tools for ArcGIS (HawthsTools® 2009), and used as sampling sites. The points were transferred to a boat-mounted Global Positioning System (GPS) unit, which was used to navigate to each study site. In case non-reef sites were selected, the field team was given alternative GPS coordinates within each habitat. More sampling effort was allocated to patch and outer reef habitats because in terms of reef area, there were disproportionally more patch and outer reefs than bays (patch reefs ∼53 km^2^, outer reefs ∼31 km^2^, and bays ∼17 km^2^). The final sampling effort examined 30 sites on the outer reefs, 30 sites on patch reefs, and 20 sites in the bays.

The survey consistently targeted the shallow coral-reef assemblages, 2–5 m below Low Water Datum (LWD). At each site we estimated the percentage coral cover by photographing 15×1 m^2^ randomly placed quadrats [37]. To estimate coral cover, the photographs were analyzed using CPCe® [38]. Ten random points were used to determine coral cover within each quadrat. The density of *Acropora, Pocillopora,* and *Stylophora* juveniles (<5 cm) was also examined in each photograph (note that *Acropora* spp., are broadcasters, and *Pocillopora* and *Stylophora* are brooders). The reef density was determined by counting the total cells of the bathymetric map that were <5 m LWD, which were considered reef area. The bathymetric map of the main islands of Palau was used for the hydrodynamic model. The grid size of the bathymetric map is 500 meters. Reef density is the fraction of the area covered by reefs. It is a different parameter than coral cover, which is the fraction of the reef substrate covered by live coral. Reef density is a bathymetric parameter, whereas percentage coral cover is an ecological parameter.

### Modeling and Data Analysis

The basis of the coral-larval oceanographic model was the finite-difference, implicit, 2-dimensional, advection-dispersion model of Wolanski and King [39]. The model incorporated the currents driven by swell waves breaking on a reef flat – parameterized as in Wolanski et al. [340]. The model domain was a rectangle 127 km by 176 km with a grid size of 500 m, set at a maximum depth of 80 m in oceanic waters, to represent the surface-mixed layer, and setup with 4 open boundaries in the ocean (Figure 3). To prevent instabilities, a 10-cell wide sponge layer was added to the western open boundary to prevent the reflection of outward-going waves back into the model domain; the sponge absorbed high-frequency surface waves but did not disturb the tidal and low-frequency currents. The model also incorporated a uniform wind forcing. The tides η were specified on the western boundary (boundary 1). The north/south current U (>0 if northward, <0 if southward) was specified both on the northern boundary (boundary 2) and southern boundary (boundary 4), and the east/west current V (>0 if eastward, <0 if westward) was specified on the eastern boundary (boundary 3) (Figure 3). The open boundary conditions were set from field observations. The tides, η, at open boundary 1 were provided by NOAA. The currents at the three other open boundaries were set from field data using:
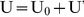
(1)where U_o_ is the low-frequency oceanic current in the far field upstream of Palau, and U’ is the tidal current, and similarly,
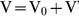
(2)In other words, the tidal current velocities were added to the far-field low frequency current velocities.The tidal currents (U’, V’) were taken from current data obtained using a Sontek Acoustic Doppler Current Profiler (ADCP) attached for a month at 20 m depth to a Fish Aggregation Device, about 9 km offshore from central eastern Babeldaob in 3000 m depth (Figure 1). The low-frequency currents (U_o_,V_o_) were taken from altimetry data (http://www.oscar.noaa.gov) and averaged from 1992–2009, calculated in a 1×1 degree area located just to the east of Palau. To execute the model we imposed a 1 m easterly swell, 18 km hr^−1^ northeasterly winds, and a 1.6 m tidal range. These conditions are based on data from Palau's National Weather Service station for the spawning period in March/April, averaged over the past 3 years.

The altimetry data was also used to examine whether water-borne larvae from coral reefs around Yap could drift to Palau, and how long that would take. Yap is located about 400 km ‘upstream’, to the northeast, of Palau. The likely recruitment rate during such long-distance events is much lower than that expected from local recruitment, primarily because mixing and diffusion at sea reduces larvae concentrations considerably. Therefore dilutions were calculated using Fischer et al. [41] 2-D Fickian diffusion model.

The advection-dispersion model was Lagrangian and follows Spagnol et al. [42]. The residence time of waters in the archipelago is determined not just by the mean circulation but also by diffusion. The model accounts for mixing at scales larger than the grid size (500 m). Sub-grid size diffusion was parameterized by an eddy diffusion coefficient of 1 m^2^ s^−1^, a value recommended by Okubo [41] for mixing of patches of that size, and implemented numerically using a random Markov walk [40].

Fifty evenly spaced spawning sites were selected within the model domain. Each site consisted of a square of 3×3 cells of the model, which translated into a 1500 m by 1500 m area. Nine cells was necessary to smooth over the inherent complexities of variable velocity fields within reef systems; near stagnant cells occasionally occur adjacent to cells with high velocity [44]. Five-thousand larvae were released from each cell (45,000 larvae from each site) over 30 minutes and tracked for 120 hours. We ran the model for more than 120 hours in consideration of the ∼ 5 day pre-competency period of most coral larvae. These numbers were standardized to examine local retention and dispersion patterns. The numbers are not meant to represent larvae densities in the field, because one large *Acropora* colony can release thousands of gametes simultaneously. We calculated: (i) the self-seeding of reefs, i.e. the percentage of larvae particles remaining in the site after 120 hours; (ii) the connectivity of reefs, i.e. the number of larvae at each site that were derived from other release sites after 120 hours, and (iii) the total seeding of larvae, i.e. the sum of larvae retained at a given site and the larval imports from other release sites after 120 hours.

These data were used to calculate and plot the spatial distribution of the self-seeding of reefs, and the total seeding of larvae using ordinary kriging analysis on the ArcGIS platform using Gaussian functions for best-fit modeling [45]. One of the assumptions of kriging analysis is stationarity, or steady state of the system. There was mass mortality of corals in Palau following the 1997–1998 El Niño, yet the coral cover recovered and reached a steady state around 2007. Moreover, such stationarity implies that a numerical model is justified to quantify the relationship between sources and sinks of coral larvae in Palau at present.

We used regression analyses to compare the outputs of the dispersal model, which included the variables: (i) the percentage of larval retention (i.e., self-seeding), (ii) the number of larvae from other sites, and (iii) the total number of larvae at each site. These variables were then correlated with the percentage hard coral cover. We also used Spearman’s rank correlation to compare the outputs of the dispersal model with the densities of juvenile coral colonies. To check for spatial autocorrelation problems we ran a Moran’s I test in R (R Core development team, 2011, version 2.1) on a windows platform, treating the latitude and longitude values on a plane, rather than on a sphere, because the spatial scale of this analysis did not extend beyond 1^o^ in latitude.
